# Highly-cited estimates of the cumulative incidence and recurrence of vulvovaginal candidiasis are inadequately documented

**DOI:** 10.1186/1472-6874-14-43

**Published:** 2014-03-10

**Authors:** Sujit D Rathod, Patricia A Buffler

**Affiliations:** 1 Division of Epidemiology, University of California, Berkeley, USA; 2 Department of Population Health, London School of Hygiene and Tropical Medicine, London, UK

## Abstract

**Background:**

Available literature concerning the epidemiologic or clinical features of vulvovaginal candidiasis commonly reports that: 75% of women will experience an episode of vulvovaginal candidiasis in their lifetimes, 50% of whom will experience at least a second episode, and 5-10% of all women will experience recurrent vulvovaginal candidiasis (≥4 episodes/1 year). In this debate we traced the three commonly cited statistics to their presumed origins.

**Discussion:**

It is apparent that these figures were inadequately documented and lacked supporting epidemiologic evidence. Population-based studies are needed to make reliable estimates of the lifetime risk of vulvovaginal candidiasis and the proportion of women who experience recurrent candidiasis.

**Summary:**

The extent to which vulvovaginal candidiasis is a source of population-level morbidity remains uncertain.

## Background

Vulvovaginal candidiasis is a commonly reported gynecological condition and is diagnosed in a large proportion of women presenting to medical facilities with a complaint of abnormal vaginal discharge [1]. While not a cause of mortality, the morbidity associated with vulvovaginal candidiasis make it a major cause of mental distress [2] and economic costs [3]. Though there are well-recognized limitations of the existing epidemiologic data for vulvovaginal candidiasis [4], frequently-cited incidence and recurrence figures reported in the vulvovaginal candidiasis literature are not, in fact, supported by published epidemiologic studies. Specifically, the literature describing the epidemiologic and clinical features of vulvovaginal candidiasis commonly reports that approximately:

•75% of women will experience an episode of vulvovaginal candidiasis in their lifetimes [5-56],

•50% of initially infected women will experience at least a second episode [5,6,8,10,11,13-15,18,20],[22-25,27,29-33,36-40,44,46,50,51],[53], and

•5-10% of all women experience recurrent vulvovaginal candidiasis (RVVC) (≥4 episodes/1 year) [6,9,11,16,18,21,23-26,28],[29,31-34,39,43-45,48,51-53,57-62].

An investigation into the sources of these statistics suggests that these commonly reported figures ultimately represent restatements of information derived from unpublished reports and clinical opinion. The regular reference to these undocumented estimates in the literature has developed an “unfounded authority”, which masks the need for further study of the epidemiologic features of vulvovaginal candidiasis.

## Discussion

While searching for the primary studies that would provide the epidemiologic support for these statistics, we noted that a number of articles in gynecology journals either directly, or indirectly via intermediary articles, refer to the work of Hurley, with the assertion that, “*75*% *of women will experience an episode of vulvovaginal candidiasis in their lifetimes*.” In 1977, Hurley provided a historical overview of *Candida* vaginitis at a meeting of experts from the United Kingdom and Belgium [63]. Hurley referred to work by Ajello, stating that “*the true incidence and prevalence of mycotic disease remains unknown*” [64]. In a later publication, Hurley and de Louvois reported that “*It is likely that between 1*/*20 to 1*/*7 of women of child*-*bearing years suffer from* Candida *vaginitis*” [65]. It is clear from reading Hurley’s cited work that she did not purport to estimate the lifetime incidence of vulvovaginal candidiasis, yet her publications are frequently cited by others to support the 75% figure.

With regard to the estimate of repeat episodes of vulvovaginal candidiasis experienced by women, Hurley is again directly or indirectly cited by others to support the assertion that: “*50*% *of those women will experience at least a second episode* [*of vulvovaginal candidiasis*].” Our search for the source of this estimate indicates that the likely source is a presumably unpublished study Hurley describes in a paper published in 1977: “*A retrospective survey of some 500 women treated for pregnancy thrush showed that 45*% *had had more than one course of treatment during pregnancy*” [63]. Hurley does not provide a reference for this study. Hurley refers to similar results from “unpublished observations” in a paper published in 1975: "*A retrospective survey* (*Hurley and Stanley*, *1973*) *showed that more than half of 300 women treated for pregnancy thrush had had two or more courses of therapy*” [2]. The studies to which Hurley refers concern treatment failure – i.e. not of multiple, distinct episodes of vulvovaginal candidiasis - in women during pregnancy, and apparently these estimates were never published in the peer-reviewed literature. Thus, the citations attributed to Hurley do not provide adequate documentation for the 50% recurrence estimate.

There also does not appear to be adequate support for the statement: “*5*-*10*% *of all women experience recurrent vulvovaginal candidiasis*.” The earliest reference to this estimate was traced to Sobel, who in 1993 stated that: “*A small subpopulation of undetermined size*, *probably less than 5*% *of adult women*, *has recurrent*, *often intractable*, *episodes of this disorder*” [18]. The previous year Sobel specifically stated “*There are no accurate figures describing the magnitude of the group with recurrent infection*”, which he found was still the case in 2003 “*The true incidence of RVVC remains unknown*” [66,67]. Though there is ample anecdotal evidence that many women suffer from recurrent vulvovaginal candidiasis, Sobel’s publications do not purport to definitively estimate the proportion of the population comprised of these women. Since Sobel’s estimate was published there have been two population-based studies of the prevalence of recurrent vulvovaginal candidiasis, both of which found that approximately 8% of women are affected by RVVC [3,68]. These studies – though the most rigorous available – must be interpreted with caution, as few of the women recruited opted to participate, there was evidence that recall of past diagnoses diminished over time, and the criteria the participants’ physicians used to make the diagnoses were unknown. Though a co-author on these studies, Sobel himself has remarked that the use of self-reported recall of physician diagnoses “multiplies errors”, and that other studies are subject to selection bias in the form of women self-selecting to become patients [4].

A limiting characteristic of other studies cited with regard to the cumulative incidence of vulvovaginal candidiasis is the use of self-reported history of vulvovaginal candidiasis [3,13,68-72]. For example, Berg noted that 72% of 204 adult women visiting a medical center in the United States reported a history of yeast infections [69]. Yet, few authors subsequently citing Berg note the self-reported nature of these data. In their review of genital candidiasis, Achkar and Fries found only two population-based studies of the incidence of vulvovaginal candidiasis, both of which relied on self-reported diagnoses [5]. In the absence of laboratory-confirmation of *Candida* in women with vulvovaginal candidiasis-associated symptoms, both self-diagnosis and clinical diagnosis are known to be of low accuracy [4,73-76].

Accordingly, diagnostic guidelines from the US Centers of Disease Control recommend use of wet mount, culture or other laboratory tests to confirm the presence of vaginal *Candida* among women reporting symptoms consistent with vulvovaginal candidiasis [51]. Conversely, population-based studies which only measure vaginal colonization by *Candida* without clinical examination cannot confirm diagnoses of symptomatic vulvovaginal candidiasis [74]. While it is valuable to understand the incidence of vaginitis, and of vaginal carriage of *Candida*, neither alone is sufficient to estimate the cumulative incidence or recurrence of vulvovaginal candidiasis.

The peer-reviewed literature on vulvovaginal candidiasis continues to report these unsupported estimates for the incidence and recurrence among all women. One may posit that over time these estimates have fostered an impression among clinicians that women with vaginitis have a high probability of having vulvovaginal candidiasis, so much so that many clinicians dispense with confirmatory tests and rely on syndromic diagnoses. Then, in the words of Sobel: “Misdiagnosis by clinicians inevitably results in incorrect self-diagnosis by patients” [4] – a finding which has been confirmed [77]. These misdiagnoses will continue to manifest themselves in research data which rely on self-reported recall of self- or physician-diagnosed vulvovaginal candidiasis.

Researchers who publish reports concerning vulvovaginal candidiasis continue to cite the publications of Hurley and Sobel. Based on our inquiry into the origins of these estimates in the vulvovaginal candidiasis literature, it appears that these inadequately documented estimates have acquired an “unfounded authority” via repeated citation in the peer-reviewed literature, a phenomena described by Greenberg [78].

Population-based cohort studies are essential for providing reasonable estimates of the incidence and recurrence of vulvovaginal candidiasis. A study of this nature requires: 1) a population-based survey of women to identify prevalent infections; 2) prospective follow-up of an initially unaffected cohort for at least one year; 3) laboratory testing for the presence of *Candida* species upon report of vulvovaginal candidiasis-associated symptoms; and 4) exclusion of *Candida* as an “innocent bystander” (i.e. when symptoms are a consequence of another condition) [4]. Absent data derived in this manner, it is not possible to make reliable estimates of the incidence and recurrence of vulvovaginal candidiasis in a population.

## Summary

To obtain resources needed for the investigation of a non-lethal health condition, public health researchers must demonstrate that the condition represents a substantial burden on a population level. In the case of vulvovaginal candidiasis, this is contingent on vulvovaginal candidiasis having an unacceptably high incidence and recurrence. As described above, the commonly cited incidence and recurrence figures were not derived from empiric investigation. Thus, the extent to which vulvovaginal candidiasis is a source of population-level morbidity remains uncertain.

## Competing interests

The authors declare that they have no competing interests.

## Authors’ contributions

SDR conceived and designed the review of the literature, drafted the manuscript, and revised the manuscript for resubmission. PAB revised the drafts critically for important intellectual content. Both authors analysed and interpreted the results, and have given final approval of the version to be initially submitted.

## Pre-publication history

The pre-publication history for this paper can be accessed here:

http://www.biomedcentral.com/1472-6874/14/43/prepub
